# The Acute Host-Response of Turkeys Colonized With *Campylobacter coli*

**DOI:** 10.3389/fvets.2021.613203

**Published:** 2021-04-06

**Authors:** Matthew J. Sylte, Sathesh K. Sivasankaran, Julian Trachsel, Yuko Sato, Zuowei Wu, Timothy A. Johnson, Lawrance C. Chandra, Qijing Zhang, Torey Looft

**Affiliations:** ^1^Food Safety and Enteric Pathogens Research Unit, National Animal Disease Center, Agricultural Research Services, U.S. Department of Agriculture, Ames, IA, United States; ^2^Genome Informatics Facility, Iowa State University, Ames, IA, United States; ^3^Veterinary Diagnostic Laboratory, Iowa State University, Ames, IA, United States; ^4^Department of Veterinary Microbiology and Preventive Medicine, Iowa State University, Ames, IA, United States

**Keywords:** *Meleagris gallopavo* (Turkey), *Campylobacter coli*, cecal tonsil, RNAseq analysis, acute phase protein

## Abstract

Consumption of contaminated poultry products is one of the main sources of human campylobacteriosis, of which *Campylobacter jejuni* subsp. *jejuni* (*C. jejuni*) and *C. coli* are responsible for ~98% of the cases. In turkeys, the ceca are an important anatomical site where *Campylobacter* asymptomatically colonizes. We previously demonstrated that commercial turkey poults colonized by *C. jejuni* showed acute changes in cytokine gene expression profiles, and histological intestinal lesions at 2 days post-inoculation (dpi). Cecal tonsils (CT) are an important part of the gastrointestinal-associated lymphoid tissue that surveil material passing in and out of the ceca, and generate immune responses against intestinal pathogens. The CT immune response toward *Campylobacter* remains unknown. In this study, we generated a kanamycin-resistant *C. coli* construct (CcK) to facilitate its enumeration from cecal contents after experimental challenge. *In vitro* analysis of CcK demonstrated no changes in motility when compared to the parent isolate. Poults were inoculated by oral gavage with CcK (5 × 10^7^ colony forming units) or sterile-media (mock-colonized), and euthanized at 1 and 3 dpi. At both time points, CcK was recovered from cecal contents, but not from the mock-colonized group. As a marker of acute inflammation, serum alpha-1 acid glycoprotein was significantly elevated at 3 dpi in CcK inoculated poults compared to mock-infected samples. Significant histological lesions were detected in cecal and CT tissues of CcK colonized poults at 1 and 3 dpi, respectively. RNAseq analysis identified 250 differentially expressed genes (DEG) in CT from CcK colonized poults at 3 dpi, of which 194 were upregulated and 56 were downregulated. From the DEG, 9 significantly enriched biological pathways were identified, including platelet aggregation, response to oxidative stress and negative regulation of oxidative stress-induced intrinsic apoptotic signaling pathway. These data suggest that *C. coli* induced an acute inflammatory response in the intestinal tract of poults, and that platelet aggregation and oxidative stress in the CT may affect the turkey's ability to resist *Campylobacter* colonization. These findings will help to develop and test *Campylobacter* mitigation strategies to promote food safety in commercial turkeys.

## Introduction

Campylobacteriosis is the most prevalent bacterial foodborne disease in humans worldwide (1). Thermophillic *Campylobacter jejuni* subsp. *jejuni* (*C. jejuni)* and *C. coli* are responsible for almost 100% of cases in humans, of which up to 70% of human cases (2) are caused by ingesting or handling contaminated poultry products (3). As intestinal commensals, thermophilic campylobacters asymptomatically persistently colonize the cecum of chickens (4) and turkeys (5, 6). Acutely after colonization, some strains of *C. jejuni* have been shown to induce an upregulation of pro-inflammatory cytokines and chemokines genes in the cecum of chickens (7–13) or turkeys (6), as well as mild histological changes were detected in the cecum of turkeys post-colonization (6). In turkeys, expression of pro-inflammatory genes and histologic lesions resolved by day 7 post-colonization to levels detected in mock-colonized age-matched poults (6). Similar kinetics in the expression of pro-inflammatory cytokine interferon-gamma (IFNγ) were detected in cecal tissue of chickens colonized by *Campylobacter* (7). It appears that *Campylobacter* can modulate the cecal immune response shortly after colonization, but the mechanism is unknown. Studying the acute host-response may help design mitigation strategies to diminish *Campylobacter* colonization in turkeys.

Both chickens and turkeys lack intestinal lymph nodes, but have a diverse complement of gastrointestinal-associated lymphoid tissues (GALT) populating the mucosal surface of the intestinal tract, and include the bursa of Fabricius, cecal tonsils (CT), Peyer's patches, Meckel's diverticulum, and discreet lymphocyte aggregates located in the intestinal lamina propria. Poultry have two ceca, each with an accompanying CT. Anatomically, CT are uniquely situated at the base of each cecum, where immune cells sample intestinal contents entering and leaving each cecum. Intestinal contents are propelled in between the ceca and colon of poultry in a process of peristalsis and antiperistalsis, which is controlled by the enteric nervous system (14). *C. jejuni* heavily colonizes the cecum of turkeys with ≥10^8^ cfu/g of cecal contents after experimental inoculation (5). The CT are large lymphoid aggregates in the poultry GALT, and are important to control infectious diseases or respond to vaccines in the distal intestinal tract (15, 16). The cellular composition of chicken CT are rich in B and T cells (17), and IgA secreting cells (18) and CD4^+^ T cells (19) in turkeys. Initially, CD4^+^ T cells populate the chicken CT, and are slowly replaced in time by B cells and IgM/IgA secreting plasma cells (20). In spite of repeated exposure of *Campylobacter* to cells of CT, little is known about the role of CT immune response to *Campylobacter*, especially *C. coli*. Germ-free chicks mono-associated with *C. jejuni* demonstrated an influx of B and T cells to the CT (21). Differences in the expression of pro-inflammatory cytokines and chemokines were detected in chicken CT tissue following oral gavage with a *C. jejuni* lysate, with or without different adjuvants (22). *C. jejuni* outer membrane proteins can stimulate an inflammatory response after directly interacting with CT cells (23). In this study, turkeys were acutely colonized with *C. coli* and we sought to characterize the host-response in intestinal tissues, focusing on the CT. We hypothesized that changes in gene expression in the CT of *C. Coli* colonized poults may help explain why poults fail to clear *Campylobacter* after inoculation.

## Materials and Methods

### Generation of *Campylobacter coli* Antibiotic Resistant Construct

The *CmeF* locus was selected as insertion site of kanamycin (Kan) antibiotic resistant cassette into wild-type *Campylobacter coli* strain 80-102 (ATCC 43481), which was originally isolated from turkey feces in Colorado. This locus was previously used to transform wild-type *C. jejuni* strain NCTC 11168 with chloramphenicol or kanamycin resistance cassettes to create antibiotic resistant constructs (6, 24). Briefly, two sets of primers were designed to amplify both ends of the *CmeF* gene and were used as the flanking sequences to help integrate the Kan resistance cassette into the *C. coli* chromosome by homogenous recombination (Pair one: Cme-1F: 5′-CCTAAGGAAAGATCATTCACTCCAGCTGTG-3′, Cme-1R:5′-GATATATTGATAAGCGGGATCCGCGTGCAGGCATTGATGATCCCG-3′; Pair two: Cme-2F:5′-GTCTTAGCATTATCCTGCAGTTCGCAGCTTGTAAAGGCGGAT-3′, Cme-2R: 5′-GAACTTAGCAATCTTCGCATAAAAACAGGAG-3′). The Kan resistance cassette was amplified from pMW10 (25) by the following primers: kanF 5′-CGCGGATCCCGCTTATCAATATATCTATAGAATGG-3′, kanR 5′-GAACTGCAGGATAATGCTAAGACAATCACTAAAG-3′. An overlap PCR was performed to get a three-fragment-ligated product (CmeF-part1_Kan_CmeF-part2). The CmeF-Kan resistance cassette fragments were introduced into *C. coli* isolate 80-102 using an electroporator (Gene Pulser Xcell System; Bio-Rad Laboratories, Richmond, CA, USA), and incubated onn Muller Hinton agar (MH; Neogen Corporation, Lansing, MI) for 5 h. Transformants (*CmeF*::*Kan*) were selected on MH agar containing kanamycin (30 μg/L) cultured at 42°C in a microaerophilic gas environment (5% O_2_, 10% CO_2_ and 85% N_2_ gas) for 48 h, and individual colonies were tested using PCR to ensure the Kan resistance cassette was inserted into the *cmeF* gene. The resistance of different *CmeF*::*Kan* transformants was determined by culturing individual colonies were on Campy Line agar (CLA) (26) containing different concentration of kanamycin (25, 50 or 100 μg/mL) at 42°C in a microaerpohillic gas environment. *CmeF*::*Kan* transformants (herein named CcK) were considered kanamycin resistant if they grew on CLA containing at least 100 μg/mL kanamycin. Once antibiotic resistance was determined, aliquots of the strain were stored at −80°C in MH broth containing 10% (v/v) sterile glycerol.

### *C. coli* Motility and Growth Curves

The motility of wild type *C. coli* or kanamycin-resistant construct CcK used in these studies were assessed each time it was used. Briefly, cryopreserved *C. coli* were inoculated onto a CLA plate, with or without kanamycin (100 μg/mL), and were cultured in a microaerophilic gas incubator at 42°C for 18 h. Up to 5 colony forming units (cfu) of each strain were used to inoculate Bolton's broth base (Neogen Corporation), and was incubated shaking (100 rpm) in a miroaerophillic gas environment at 42°C for 18 h. Ten microliter of each broth culture was microscopically visualized at 400X magnification using a Nikon Eclipse Ni dark-field microscope (Nikon Instruments Inc., Melville, NY). Motility was visually assessed. Strains were considered motile if they had a positive motility agar test or had at least 90% of the organisms actively moving. If motility wasn't detected in the agar stab, or <90% were motile by microscopic observation, the inoculum was not used.

Preparation of the growth curve inocula were performed by static culture of CcK and its parent strain at 42°C in the broth phase of biphasic MH broth and agar (2% w/v) in a microaerophilic gas environment (5). Growth curves were performed in octuplet using instrument-specific microplates in a Bioscreen C plate reader (Growth Curves USA, Piscataway, NJ), measuring OD_600_ every 2 h for 48 h. To limit aggregation, which may impact optical density values, microplates were shaken for 30 s prior to each OD_600_ reading. For each replicate of each isolate, MH broth culture volumes were 200 μL, and were incubated at 42°C in a microaerophilic gas chamber. Uninoculated media served as a control to subtract OD_600_ background. The logistical area under the curve, growth rate and generation time were determined by analyzing growth curve data using the R package growthcurver (27).

### Animal Experimental Design

This animal experiment was conducted according to the regulations established by the NADC Institutional Animal Care and Use Committee. Day of hatch Hybrid jake poults (*n* = 45) were obtained from a commercial breeder and co-housed in a single ABSL-2 room. Throughout the study, poults were fed a turkey poult starter ration and had water available *ad libitum*. The *Campylobacter* status of experimental poults was determined at day 15 of age by randomly selecting and euthanizing *n* = 5 poults. Necropsy was performed to harvest 1 g of cecal contents, which was cultured by enrichment in 10 mL of Bolton's broth base containing *Campylobacter* selection supplement (Neogen Corporation) for 48 h in a microaerophilic gas environment at 42°C (28, 29). Afterward, 100 μL was cultured for 48 h at 42°C in a microaerophilic environment on Campy Line agar containing 25 μg/mL sulfamethoxazole (CLA-S) (30). Poults were considered free of *Campylobacter* colonization if no colonies resembling pure cultures of *C. jejuni* or *C. coli* were recovered after enrichment. After co-housing for 20 days, the remaining (*n* = 40) poults were evenly distributed into two ABSL-2 rooms for challenge (*n* = 20/room). Room temperature, humidity and lighting cycle were approximately the same for the ABSL-2 rooms. Preparation of *C. coli* CcK inoculum was performed by subculturing at least 5 colonies from a pure culture of CcK on CLA-S agar supplemented with 100 μg/mL kanamycin (CLA-S-K) into 100 mL of MH broth and cultured in a microaerophilic gas environment at 42°C shaking at 100 rpm for 48 h. On the day of challenge, the OD_600_ value was adjusted to 0.4 in sterile MH broth. The cfu/mL of each inocula was enumerated using serial dilation CLA-S agar supplemented with 100 μg/mL kanamycin, and motility was assessed using dark-field microscopy, as described above. At 21 days old, all poults (*n* = 20) within a room were individually inoculated by orally gavage with either 1 mL of MH broth containing approximately 5 × 10^7^ cfu of CcK, or mock inoculated with 1 mL of sterile MH broth. At 1- and 3-days post-inoculation (dpi), *n* = 10 poults from the CcK or mock-inoculated rooms were randomly selected, bled from their brachial vein and euthanized by intravenous barbiturate overdose. Necropsy was performed to harvest cecal contents for enumeration of CcK from each animal. Intestinal tissues, including ileum, CT and colon were fixed in buffered neutral formalin for histological analysis and the remaining CT was preserved in RNALater for isolation of total RNA.

### Enumeration of *C. coli* From Intestinal Samples

Cecal contents were stored on ice and transported to the laboratory for culture. From each animal, 1 g of cecal contents was diluted in 9 mL of sterile PBS, vortexed for 5 s and serially diluted up to 10^−6^. Enumeration was performed utilizing the track-plating dilution method (5, 31), where 10 μL of each dilution was plated in duplicate on CLA-S-K and incubated at 42°C in a microaerophilic environment for 48 h. Colony forming units resembling those from pure cultures of *C. coli* isolate 80-102 were enumerated. For statistical purposes, if no colonies resembling *C. coli* grew, the sample was assigned the culture limit of detection value of 10^3^ cfu/g of contents. Poults were considered positive for *Campylobacter* colonization if at least one colony was cultured on CLS-S-K media.

### Histological Analysis

At necropsy, colon, ileum, cecum and a CT were immersed in 10% buffered neutral formalin, and fixed for 48 h. Tissues were transferred and stored in 70% ethanol before cutting into histology cassettes and embedding in paraffin for thin sectioning (5 μm), and stained with hematoxalin and eosin. Slides were single-blinded analyzed and scored by a poultry pathologist (YS) for lesions. Because *Campylobacter* are commensals of the poultry intestinal microbiota, and severe lesions are not observed in experimentally colonized poults (6), a unique scoring system was used to evaluate changes of the intestinal histomorphology. The following criteria were scored from ten random fields per tissue at 400X magnification: (1) Heterophils in lamina propria; score (<3, 0; ≥3–5, 1; ≥5–10, 2; ≥10, 3), (2) Heterophil margination in epithelium; score (<10%, 0; ≥10–25%, 1; ≥25–75%, 2; ≥75%, 3), (3) Edema in lamina propria; score (<10%, 0; ≥10–25, 1; ≥25–75, 2; ≥75, 3), (4) Goblet cell hyperplasia; score (<10%, 0; ≥10–25%, 1; ≥=25–75%, 2; ≥75%, 3). (5) Crypt ectasia (small) <100 μm; score (<3, 1; ≥3–5, 2; >5, 3), (6) Crypt ectasia (large); ≥100 = 400 μm, add 2 to score from crypt ectasia (small); ≥400 μm, add 3 to score), (7) Apoptotic/necrotic cells in lamina propria; score (<10%, 0; ≥10–25%, 1; ≥25–75%, 2; ≥75%, 3) and (8) Blunting ± fusion of epithelium; score (no lesions, 0; mild blunting, 1; moderate to marked blunting, 2; severe blunting and fusion ± blebbing, 3). For each group, the final scores were averaged. The minimal and maximal scores were 0 and 24, respectively.

### Alpha-1 Acid Glycoprotein ELISA

Prior to euthanasia, ~3 mL of blood was sampled from the brachial vein and clotted by incubating at 37°C for 4 h. Afterwards, blood was centrifuged at 1,200 × g for 10 min at 4°C, serum was removed and stored at −20°C. Serum alpha-1 acid glycoprotein (AGP) levels were measured, performed in triplicate for each serum sample, using a commercially available turkey-specific AGP ELISA (Life Diagnostics Inc.; West Chester, PA). Following the manufacturer's protocol, serum samples were diluted and absorbance at 405 nm was measured using a Synergy HT spectrophotometer (BioTek; Wintooski, VT). Per the manufacturer's recommendation, the AGP concentration (ng/mL) was extrapolated from an AGP standard curve, using BioTek Gen 5 software.

### RNA Preservation, Total RNA Extraction, and RNAseq Library Preparation

Total RNA from the remaining CT from each poult was preserved by immersing the tissue in 5 mL of RNALater stabilizer solution (Life Technologies, Carlsbad, CA), and were incubated at 4°C for 24 h (6). The CT was snap frozen by placing it in an internally threaded cryovial and fully immersing in liquid nitrogen for 1 min. Cryovials were then stored indefinitely in a −80°C freezer. For total RNA isolation, up to 150 mg of CT was placed into a gentleMACS M tube (Miltenyi Biotec Inc., San Diego, CA) containing 1 mL of TRIZOL reagent (Life Technologies). Tissue was homogenized using a gentleMACS Octo Dissociator (Miltenyi Biotec Inc.) using the gentleMACS program RNA_02. The homogenate was centrifuged in the M tube for 30 s at 800 × g at room temperature, and supernatant was transferred to nuclease-free 1.5 mL microfuge tubes. Two hundred microliter of chloroform was added and up to 300 μL of the aqueous phase was harvested after centrifugation for 15 min at 12,000 × g at 4°C. The aqueous phase was further processed using mirVana miRNA isolation kit without phenol, per the manufacturer's protocol (Ambion, Carlsbad, CA). The quantity of eluted total RNA was spectrophotometrically estimated using a NanoDrop-2000 instrument (Thermo Fisher Scientific, Waltham, MA). Per the manufacturer's protocol, up to 500 ng of total RNA was evaluated for RNA integrity using an RNA ScreenTape and 2200 TapeStation instrument (Agilent Technologies Inc., Santa Clara, CA). Total RNA was stored at −80°C in nuclease free tubes until used to prepare RNAseq libraries. All samples had RNA integrity number equivalent (RINe) values ≥ 8, indicating the high quality of total RNA isolated from CT samples. Up to 100 ng of total RNA was used to generate sequencing libraries using TruSeq stranded mRNA library kit (Illumina; San Diego, CA).

### RNAseq Analysis

Eight library samples per lane were randomly assigned across 4 lanes of a HiSeq 3000 (Illumina), and paired end 100 bp sequencing was performed. The quality of the sequenced reads was analyzed using FastQC (https://www.bioinformatics.babraham.ac.uk/projects/fastqc/). Reads were aligned to the turkey reference genome (UMD 5.0, NCBI Annotation 101) and read counts were quantified using the STAR algorithm v2.5.2b (32). Genes that had raw read count <10 in all samples were considered as a low signal and removed from the differential gene expression analysis. The gene counts for each sample were transformed and normalized using the Bioconductor package DESeq2 v1.24.0 (33) in R v3.5.0. Principle Component Analysis (PCA) was performed to determine any expression outliers based on overall gene expression counts. Linear regression models within DESeq2 were used to test differentially expressed genes between case and control groups. The effect of known covariates between case-control status including RNA quality (e.g., RNA integrity number) and sequencing slide differences was tested using Student's *T*-test (R software). In addition, to account for the underlying unmeasured confounding factors in the RNA-Seq data, we used SVAseq (34) with default parameters to estimate surrogate variables (SVs). None of the estimated SVs differed significantly between case-control status in both 1 and 3 dpi, so we did not include them in the differential gene expression analysis model. We considered fold change ≥1.5 (≥0.58 log_2_FC) with FDR < 0.05 as a significantly differentially expressed gene (DEG). To identify functional groups from the DEG, we tested gene sets of Gene Ontology Biological Processes (GO:BP) terms using DAVID (35) online tool with default parameters. The GO terms with FDR < 0.1 were considered as enriched. Raw sequenced reads and processed data have been deposited in NCBI Gene Expression Omnibus database and are available under accession GSE158639 (https://www.ncbi.nlm.nih.gov/geo/query/acc.cgi?acc=GSE158639).

### Statistical Analysis

Data for *C. coli* enumeration were analyzed using an unpaired *t*-test with Welch's correction using Prism statistical software v8.1.2 (Graph Pad Software Inc., San Diego, CA) to detect differences between time points. Data for serum alpha-1 acid glycoprotein concentration and histological scoring were analyzed for significant differences between groups or treatment days using a one-way ANOVA followed by Tukey *post-hoc* multiple comparisons test using Prism. Growth curve data were analyzed for logistical area under the curve, generation time and growth rate using the R package growthcurver (https://github.com/cran/growthcurver) (27). Results were considered significant at values of *p* ≤ 0.05.

## Results and Discussion

### *In vitro* Growth and Motility of Wild-Type *C. coli* and Kanamycin-Resistant Construct CcK

In order to more easily enumerate *C. coli* from cecal contents, a kanamycin-resistant construct (CcK) was generated by inserting the kanamycin-resistance cassette into *CmeF* (*CmeF:*:*Kan*) in the chromosome of *C. coli* parent strain ATCC 80-102. The same locus was previously used to generate antibiotic resistant constructs of *C. jejuni* (6, 24). The parent strain was killed by addition of 10 μg /mL of kanamycin into CLA-S agar, whereas CcK grew on CLA-S containing 25, 50 or 100 μg/mL of kanamycin. Analysis of growth between the parent strain and CcK (Supplementary Figure 1) demonstrated significant differences (*p* < 0.05) in the logistical area under curve, doubling time and growth rate (Supplementary Figure 2). Motility is essential for *Campylobacter* colonization in turkeys (Sylte, 2018). No difference in motility was detected between CcK and its parent strain. Overall, these data demonstrate that CcK construct didn't affect motility, but *in vitro* growth of CcK was significantly different (*p* < 0.05) than its parent strain. While the purpose of this study was not to compare the wild type and the antibiotic-resistant construct, it is important to acknowledge that these genetic changes could potentially affect colonization *in vivo*. For example, the acquisition of antibiotic resistance, particularly chromosomal mutations, comes at a biological cost, in the absence of antibiotic selective pressure. This may result in a decreased fitness such as a reduced *in vitro* growth rate or a decrease in ability to persist in the host (36–38). As such, we cannot exclude the possibility that an altered *in vitro* fitness may affect the host response compared to a wild-type *C. coli* isolate.

### Cecal Colonization of Turkey Poults With CcK

**Figure 1:**
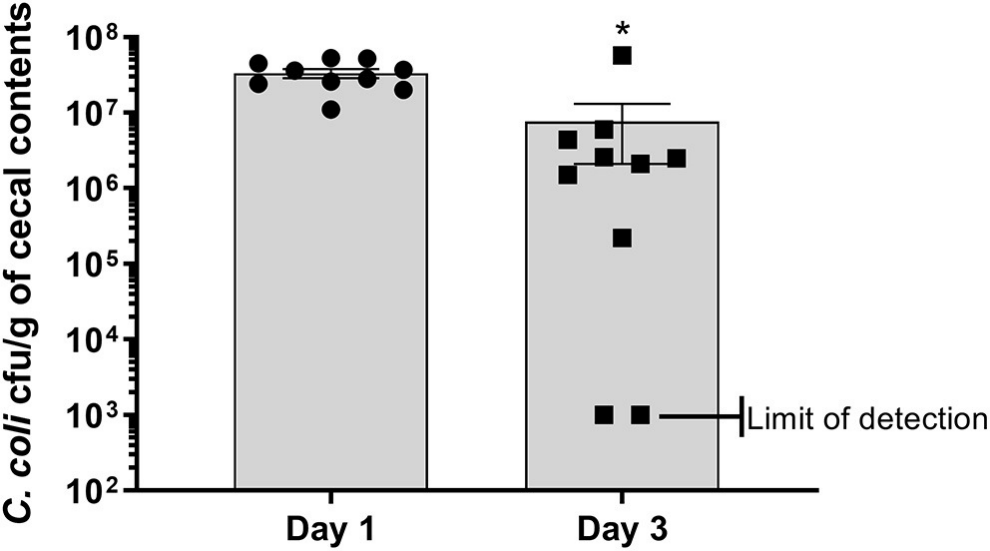
Enumeration of antibiotic resistant construct CcK from cecal contents of turkey poults. Data represent the mean CcK cfu/g of cecal contents from each poult and the mean (solid bar) ± SEM at 1 and 3 dpi. Statistical differences in the number of enumerated CcK cfu/g of cecal contents was determined using an unpaired *t*-test with Welch's correction. Significant differences (*p* < 0.05) between different time points are represented by an asterisk (*).

Poults used in this study were free of detectable *Campylobacter* prior to experimental inoculation, based on lack of *Campylobacter* recovery after enrichment of cecal contents. At 1 dpi, all sampled poults (*n* = 10) in the CcK group were positive for colonization with 3.32 × 10^7^ ± 4.4 × 10^6^ cfu/g of cecal contents (Figure 1 and Table 1). By 3 dpi, 8/10 poults (Table 1) in the CcK group were positive for colonization with significantly less (*p* = 0.0021) CcK recovered (7.63 × 10^6^ ± 5.52 × 10^6^ cfu/g of cecal contents) that at 1 dpi. Mock-colonized poults remained free of detectable CcK contamination throughout the study (Table 1). The 1 and 3 dpi CcK cecal colonization data are similar to levels seen 2 dpi in turkeys infected with chloramphenicol- or kanamycin-resistant constructs of *C. jejuni* (6). In the absence of kanamycin, a loss of fitness may explain the significant decrease in CcK cecal colonization between 1 and 3 dpi. However, we were concerned about the detrimental effects on the intestinal microbiota, and didn't administer kanamycin to poults after inoculation. Diminished fitness of *C. jejuni* in the absence of a positive selection antibiotic is supported by previous work where erythromycin resistant *C. jejuni* was less ecologically fit than the erythromycin sensitive parent strains, and failed to transmit to chickens already colonized by erythromycin sensitive *C. jejuni* (38).We think a decrease in CcK fitness would be minimized by the acute design of this study, but should be considered for animal studies longer designed to achieve *Campylobacter* persistence.

**Table 1:** Summary of CcK direct plate enumeration and post-culture validation from cecal samples 1 and 3 dpi.

**Days post- inoculation**	**Inoculation**	***C. coli* colonization**	**Cecal colonization**	
			**Direct plating culture positive**	***Campylobacter* qPCR positive**
1	CcK	Yes	10/10	10/10
	Mock	No	0/10^#^	0/10
3	CcK	Yes	8/10	8/10
	Mock	No	0/10^#^	0/10

# *Below limit of detection (10^3^ cfu/g of contents)*.

### Host-Response to CcK Colonization

**Figure 2:**
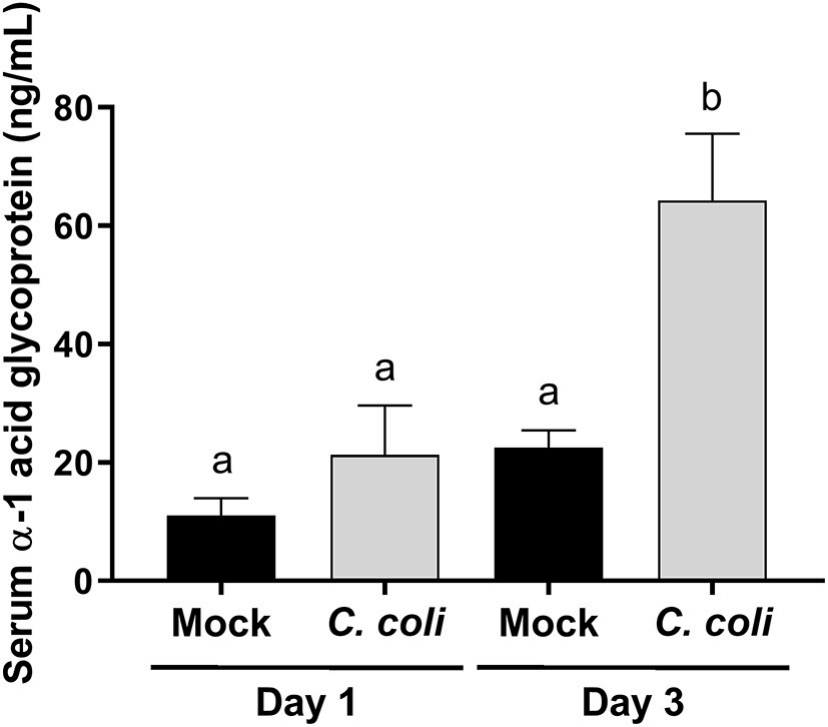
Serum alpha-1 acid glycoprotein (AGP) concentration in CcK- or mock-colonized turkey poults. Data represent the mean AGP (ng/mL) from CcK- or mock-colonized poults and the mean (solid bar) ± SEM at 1 and 3 dpi. Statistical differences between means were determined using one-way ANOVA followed by a *post-hoc* multiple comparisons test (Tukey). Significant differences (*p* < 0.05) different time points and treatments are represented by different letters.

*Campylobacter* mainly colonize the distal intestinal of turkey poults (5), and produce a temporal change in expression of pro-inflammatory genes in the cecum and intestinal histological lesions (6), but it was unknown whether the inflammatory response extended beyond the intestinal tract. Alpha-1 acid glycoprotein (AGP; also known as orosomucoid 1) is a positive acute phase protein produced in poultry. The only commercially available, turkey-specific reagent to test production of acute phase proteins is for AGP. It is best characterized in chickens, and increases in response to bacterial, viral or other inflammatory stimuli (39–44), and may modulate phagocytosis and bacterial killing by heterophils (45). Less is known about the biology of AGP in turkeys, especially if *C. coli* colonization affects serum AGP levels. At 3 dpi, serum AGP levels were significantly elevated (*p* < 0.001) in CcK-colonized poults (Figure 2). These data indicate that inflammation from CcK colonization induced a systemic response. Because of the acute nature of this study, we were unable to follow the kinetics of serum AGP levels after CcK colonization. However, our data are similar to those in layer hens orally inoculated with *S. enterica* serovar Enteritidis, which induced a significant increase in serum AGP concentration (43). Production of AGP is temporal in chickens (44), and is likely the same in turkeys after CcK colonization. Pro-inflammatory cytokines IL-1β, TNFα or IL-6 induce AGP expression from the liver (46), the main tissue producing APG in chickens (47). Our previous work demonstrated elevated cecal *il6* expression 2 dpi in *C. jejuni* inoculated poults (6). We did not test for cytokine gene expression in cecal tissue in this study, but cecal IL-6 or other pro-inflammatory cytokines may be the cause of elevated serum AGP at 3 dpi.

**Figure 3:**
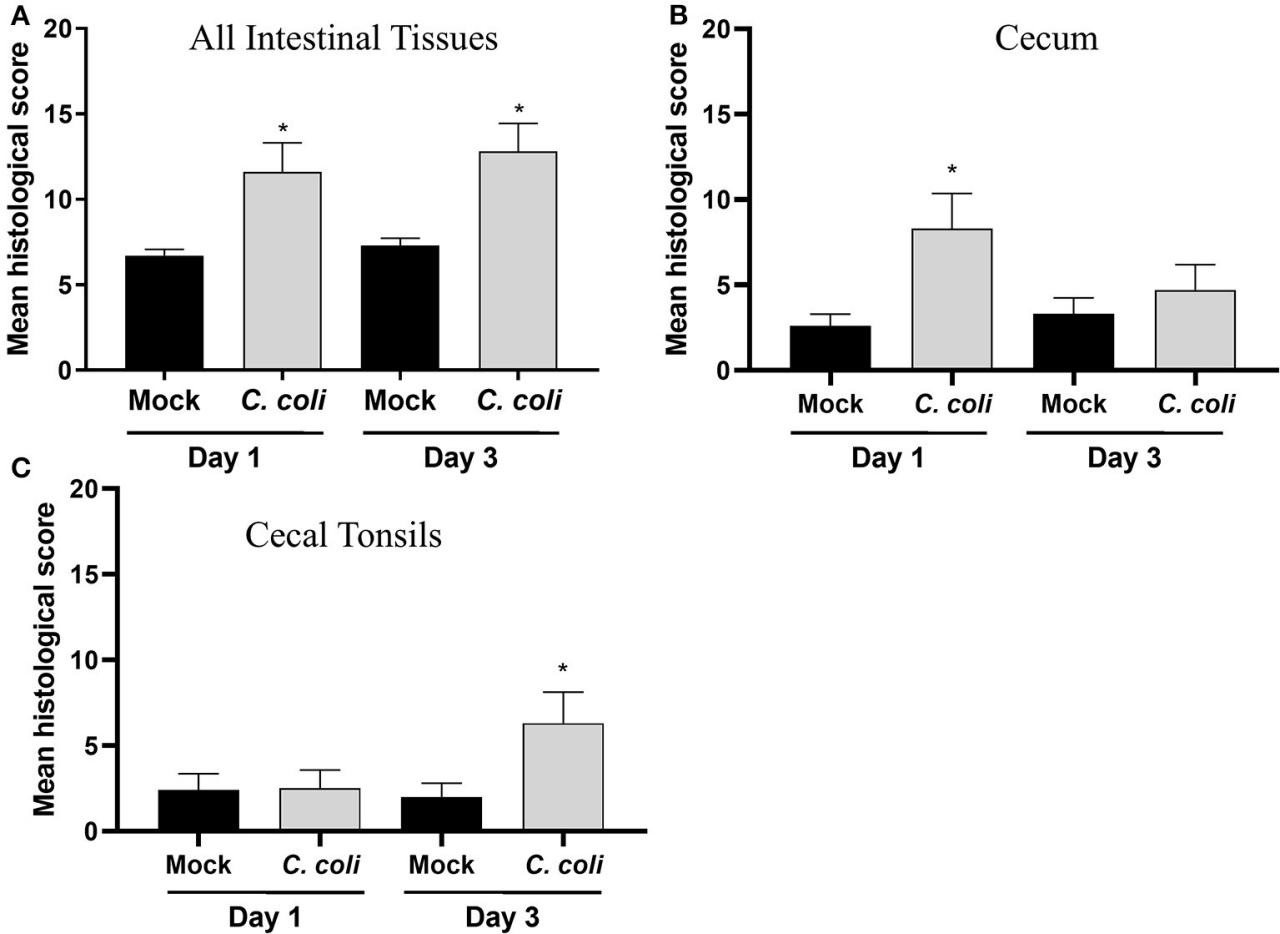
Histological scoring of intestinal tissues. A grading score was used to evaluate the following changes of the intestinal histomorphology in CcK- or mock-colonized poults at 1 and 3 dpi: (1) number of heterophils in the epithelium, lamina propria, and submucosa/muscularis as a possible indicator of inflammation and immune activation, (2) presence of small granulomas within the lamina propria, (3) crypt ectasia with or without heterophilic accumulation, (4) presence of apoptotic cells in the lamina propria, (5) areas with attenuated epithelium. Intermediate scores for each category (1–5) was obtained for all intestinal sections (ileum, cecal tonsils, ceca, and colon) for each bird. The final histological score of one poult was the sum of each intermediate scores. For each group, the final scores were averaged for **(A)** all intestinal tissues, **(B)** cecum only and **(C)** CT only and differences between means were determined using one-way ANOVA followed by a *post-hoc* multiple comparisons test (Tukey). Significant differences (*p* < 0.05) within time points are represented by an asterisk (*).

To further assess the host-response to CcK colonization, single blinded histological scoring of intestinal tissues (e.g., cecum, cecal tonsil, colon, and ileum) from mock- and CcK-inoculated poults at 1 and 3 dpi was performed. At 1 and 3 dpi, CcK inoculated intestinal tissues had significantly higher (*p* < 0.03) mean histological scores (Figure 3A). Scores for cecum and cecal tonsil tissues were analyzed, and significant lesion scores (*p* < 0.0001) were noted at 1 dpi, but not 3 dpi, in the cecum of CcK-colonized poults (Figure 3B). Significant differences (*P* < 0.0001) in histological scoring was detected at 3 dpi, but not 1 dpi, in the cecal tonsil of CcK-colonized poults (Figure 3C). Representative images for the intestinal tissue comparisons show areas of necrotic or dilated crypts with intralesional bacteria (Supplementary Figures 3–5). These results are similar to previous work where significant histological lesion scoring was detected acutely in intestinal tissues of turkeys inoculated by *C. jejuni* at 2 dpi, but not at 7 or 14 dpi (6). Common lesions in CcK inoculated cecum at 1 dpi were severe blunting of cecal villi and necrotic crypts, and degenerate heterophils in the cecal tonsils at 3 dpi.

Previously, we've demonstrated expression of pro-inflammatory genes, including IFNγ in the cecum, and histological lesions in the distal intestinal tract of poults 2 dpi *C. jejuni* colonization (6). Similar kinetics in the expression of pro-inflammatory cytokine IFNγ were detected in cecal tissue of chickens colonized by *Campylobacter*, and it's hypothesized that impairing *ifng* expression may be responsible for *Campylobacter* colonization (7). The rapid decrease in expression of pro-inflammatory genes in the cecum shortly after *Campylobacter* colonization represents an important opportunity to improve *Campylobacter* mitigation strategies. We sought to examine the acute host-response in the cecal tonsil of CcK and mock-colonized poults at 1 and 3 dpi and used RNAseq-based approaches to globally identify the transcriptional changes in the CT samples. To our knowledge, this is the first description of transcriptional changes in the CT of poultry after *Campylobacter* colonization. All CcK- and mock-inoculated samples used in this study are detailed in Supplementary Table 1. RNA integrity ranged from 9.3 to 10 for the 32 samples. At both 1 and 3 dpi, mock colonized (*n* = 8) and CcK colonized (*n* = 8) RNA samples from CT were subject to RNAseq. Using PCA analysis, 3 outliers were identified from the CcK colonized 1 dpi samples, and removed and 29 of 32 samples were processed for differential gene (DEG) expression analysis (Supplementary Figure 6). The total number of reads produced for each library ranged from 24 to 92 million with the median of 46.5 million paired-end reads per library. Approximately, 82% of sequenced reads were successfully mapped uniquely to the reference genome (Supplementary Table 1). Approximately, 13% of mapped reads failed to align due to short read length or poor read quality. A small percentage of reads were (4.4%) aligned to multiple location in the genome which were removed from the downstream analysis.

Using the criteria of fold change ≥ 1.5 and FDR < 0.05, a total of 273 DEG were identified in the acute phase (3 dpi) after CcK colonization (Supplementary Tables 2, 3), and none of the DEG were passed our filtering criteria at 1 dpi, Of the 273 DEG, 149 genes were significantly up-regulated and 124 were down-regulated. Identification of DEG at 3 dpi in CT samples agrees with CT histological lesion scores (Figure 3C). The top 10 significantly up- and down-regulated DEG are summarized in Table 2. Mal T cell differentiation protein like (MALL), was significantly downregulated at 3 dpi in CT, and functions in T cell differentiation (48). Downregulating *Mall* may impair the development of antigen-specific T cells, and promote colonization by *Campylobacter*. In poultry, food-borne pathogens, such as *Campylobacter* and *Salmonella*, may induce immunological tolerance (49, 50), which may explain why they fail to clear these bacteria. Development of regulatory T cells (Tregs) may explain how poultry become immunologically tolerant to food-borne pathogens. More cells resembling a Treg phenotype (CD4^+^, CD25^hi^, and IL-10^+^) were isolated from cecal tonsils (CT) of chickens colonized with *Salmonella enterica* serovar Typhimurium, than control chickens (51). Although we found no significant differences in expression of *il10* or other putative Treg markers in poultry (e.g., *ctla4, lag3*, and others) (19), development of Tregs by the end of this study (3 dpi) was unlikely. *Salmonella enterica* serovar Pullorum modulated CT host immunity to a Th2 response by downregulating expression of *ifng* and upregulating *il13* (52). These data, and ours, suggest that food-borne pathogens in poultry, modulate the immune response in the intestinal tract, including the cecal tonsil, of to avoid their clearance. Examining transcriptional changes at later time points may be useful to test the development of immunotolerance in the CT of *Campylobacter* colonized turkeys. *In ovo* treatment of chicken embryos with a probiotic, *Lactobacillus salvarius*, modulated the expression of CT immune genes after hatch (53). Although not attempted, it would be interesting to see whether imprinting CT gene expression with a probiotic could prevent foodborne pathogen colonization, or enhance gut health.

**Table 2:** Summary of upregulated and downregulated differently expressed genes at 3 dpi in cecal tonsil of CcK-colonized poults.

**Gene symbol**	**Fold change (log2)**	**FDR *p*-value**	**Genbank**	**Predicted gene function**
LOC100540260	−1.38	0.04579	XM_010708497	Tyrosine phosphatase receptor type transcript
LOC100545456	−1.36	0.03510	XM_010725015	Receptor accessory protein transcript variant
LOC104917316	−1.27	0.02443	XM_010728501	Homeobox protein SIX2
LOC104912995	−1.27	0.03187	XR_795065	Uncharacterized protein
LOC100546239	−1.26	0.03249	XM_010707354	LEG1 homolog-like
LOC104916149	−1.20	0.04231	XM_010727130	Homeobox protein SIX2-like
LOC104914158	−1.16	0.01238	XR_002119998	Uncharacterized protein
LOC104911266	−1.10	0.01908	XM_010712037	Iodothyronine deiodinase 3
LOC100544663	−1.04	0.03367	XM_003209101	Apolipoprotein D
LOC100540834	−1.03	0.01898	XM_010715727	Putative dimethylaniline monooxygenase [N-oxide-forming] 6
LOC104916315	−1.02	0.00257	XM_010727366	EGF-like domain-containing protein
LOC104914637	1.13	0.01804	XM_010724747	Fibrillin-1-like
LOC104912630	1.14	0.01013	XM_010716658	Collagen alpha-3(IV) chain-like
LOC104913148	1.14	0.04840	XM_019611809	Ovostatin-like
LOC100543592	1.16	0.04453	XM_003213848	Nucleoredoxin-like transcript
LOC104914991	1.17	0.01589	XR_002109873	Uncharacterized protein
LOC109364129	1.20	0.01178	XR_002110156	Uncharacterized protein
LOC104914821	1.27	0.02927	XM_019610345	PDZ domain-containing protein 2-like
LOC104912792	1.47	0.00001	XM_019619432	E3 ubiquitin-protein ligase HERC1-like
LOC104915991	1.90	0.00008	XR_002121382	Uncharacterized protein
LOC104916559	4.69	0.00035	XR_796609	Uncharacterized protein

To predict the downstream effects of the 3 dpi DEG, pathway analysis was performed using GO:BP terms using DAVID software. Using the turkey gene symbols from the list of DEG, we identified 9 BP terms (Table 3) significantly altered in 3 dpi CcK samples (FDR < 0.1). A more lenient FDR was used to maximize GO:BP terms for pathway identification, and this technique has been used to identify pathways in data sets with smaller numbers of DEG (54). Of the identified BP terms relating to inflammation, platelet aggregation has the most significant BP terms (*p* = 0.008). Platelets aggregate during hemostasis as well as during thrombosis. Histological analysis of CcK colonized tissues revealed no evidence of hemorrhage or thrombosis. Mammalian platelets are known to express pro-inflammatory cytokines, chemokines and activate inflammatory cells, but may also diminish inflammation (55). It is unknown whether *C. coli* interacts with platelets in the CT, or affects inflammation. Negative regulation of vascular permeability (*p* = 0.052) was also identified. The DEG involved in this may function to limit the formation of edema in CT after *C. coli* colonization, as well limit as an influx of inflammatory cells (e.g., heterophils). Oxidative stress and apoptosis were another common BP terms, including response to oxidative stress (*p* = 0.033), negative regulation of oxidative stress-induced intrinsic apoptotic signaling pathway (*p* = 0.044), and negative regulation of release of cytochrome C from mitochondria (*p* = 0.092). The intrinsic pathway of apoptosis is initiated by stressors such as UV radiation, oxidant stress and involves activation of caspase-9 via the release of mitochondrial cytochrome C into the cytoplasm of affected cells (56). It is unclear if these BP terms are functioning as a result of a stressor in the CT. How *Campylobacter* activate oxidant stress in the CT of colonized poults is unknown. Methionine is a key limiting amino acid in poultry, and its deficiency produced oxidant stress in CT of chickens (57). *Campylobacter* are fastidious and some strains (e.g., *C. jejuni* NCTC 11168) require methionine for growth in a minimal medium (58), and rapidly consume it from medium (59). It's not known whether the CcK isolate is similar and rapidly consumes methionine. Based on these observations, CcK may consume dietary methionine and create a deficiency in the CT to produce oxidant stress. Supplementation of dietary methionine may prevent oxidant stress in the CT after *C. coli* colonization and affect the functionality of the CT immune response.

**Table 3:** Pathway analysis: significantly enriched gene ontology (GO) biological process (BP) terms.

**Go term ID**	**BP term**	**FDR *p-*value**
GO:0070527	Platelet aggregation	0.01465
GO:0051450	Myoblast proliferation	0.03088
GO:2001214	Positive regulation of vasculogenesis	0.04095
GO:0043116	Negative regulation of vascular permeability	0.0509
GO:0006979	Response to oxidative stress	0.05896
GO:1902176	Negative regulation of oxidative stress-induced intrinsic apoptotic signaling pathway	0.06081
GO:0006412	Translation	0.07213
GO:0008299	Isoprenoid biosynthetic process	0.08983
GO:0090201	Negative regulation of release of cytochrome c from mitochondria	0.08983
GO:0048741	Skeletal muscle fiber development	0.09931

In conclusion, the results from these studies are the first to describe the host-response of the turkey CT to *C. coli* colonization. Using a *C. coli* kanamycin resistant construct CcK for ease of enumeration from cecal contents, poults were orally colonized and the acute host response was evaluated. Significant histological lesions were noted in the cecum at 1 dpi and in CT at 3 dpi, and transcriptome analysis of CT gene expression demonstrated DEG at 3 dpi. Genes involved in regulating immune function were downregulated, and pathway analysis of the DEG identified platelet aggregation, downregulation of apoptosis and decreased vascular permeability. Evaluation of gene expression at additional timepoints after colonization, with different wild type stains, would likely provide additional understanding of the dynamic responses after infection. Results from this study provide insight into host-response of the turkey CT to *Campylobacter* colonization. These findings will help to develop and test *Campylobacter* mitigation strategies to promote food safety in commercial turkeys.

## Data Availability Statement

The datasets presented in this study can be found in online repositories. The names of the repository/repositories and accession number(s) can be found in the article/Supplementary Material.

## Ethics Statement

The animal study was reviewed and approved by National Animal Disease Center Institutional Animal Care and Use Committee.

## Author Contributions

MS designed the experiment. MS, TJ, LC, and TL were involved in acquisition of the experimental data. ZW and QZ generated the antibiotic-resistant *C. coli* construct. MS, JT, SS, and YS performed data analysis and interpretation. The manuscript was drafted and revised for important intellectual content by MS, JT, SS, YS, ZW, TL, TJ, LC, and QZ, as well as, final approval of the version to be published with agreement to be accountable for all aspects of the work in ensuring that questions related to the accuracy or integrity of any part of the work are appropriately investigated and resolved. All authors contributed to the article and approved the submitted version.

## Conflict of Interest

The authors declare that the research was conducted in the absence of any commercial or financial relationships that could be construed as a potential conflict of interest.

## Supplementary Material

Supplementary Figure 1 Growth characteristics of *C. coli* wild-type (parent strain) NCTC 80-102, and kanamycin-resistant construct (CcK). Growth curves were performed in Mueller Hinton broth for 48 h at 42°C in a microaerophilic environment (5% O_2_, 10% CO_2_, and 85% N_2_). Data represent the mean ± SEM OD_600_ of 8 cultures in Bolton's broth measured every 2 h for a total of 48 h with the background value of unincolated media subtracted.

Supplementary Figure 2 Analysis of logistical area under the curve, growth rate and generation time of *C. coli* wild-type (parent) NCTC 80-102, and kanamycin-resistant construct (CcK). Growth curves were performed in Mueller Hinton broth for 48 h at 42°C in a microaerophilic environment (5% O_2_, 10% CO_2_, and 85% N_2_). Data represent the mean ± SEM logistical area under the curve, growth rate and doubling time of 8 replicate cultures in either broth. Statistical differences in the each variable were determined using the R package growthcurver followed by a *post-hoc* multiple comparisons test (Tukey). Significant differences (*p* < 0.05) between different organisms are represented by different letters.

Supplementary Figure 3 Representative Histological of ileal tissues. Representative comparisons are shown for **(A)** an ileum from a mock-colonized bird and **(B)** ileum from a CcK-colonized bird, with heterophilic ileitis marked with an arrow.

Supplementary Figure 4 Representative Histological of cecal tonsil tissues. Representative comparisons are shown for **(A)** a cecal tonsil from a mock-colonized bird and **(B)** cecal tonsil from a CcK-colonized bird, with necrotic cecal crypts with intralesional bacteria marked with a star.

Supplementary Figure 5 Representative Histological of colonic tissues. Representative comparisons are shown for **(A)** a colonic tissue from a mock-colonized bird and **(B)** colonic tissue from a CcK-colonized bird, with crypt dilatation/ectasia marked with star.

Supplementary Figure 6 PCA analysis of RNAseq samples.

Supplementary Table 1 CcK infected and mock turkey cecal tonsil RNAseq samples description and sequencing statistics.

Supplementary Table 2 List of differentially expressed genes in 1 dpi.

Supplementary Table 3 List of differentially expressed genes in 3 dpi.
