# Supplementary figures and images for: The Acute Host-Response of Turkeys Colonized With Campylobacter coli
Source: Front Vet Sci. 2021 Apr 6;8:613203. doi: 10.3389/fvets.2021.613203 (PMC8057350; doi:10.3389/fvets.2021.613203)

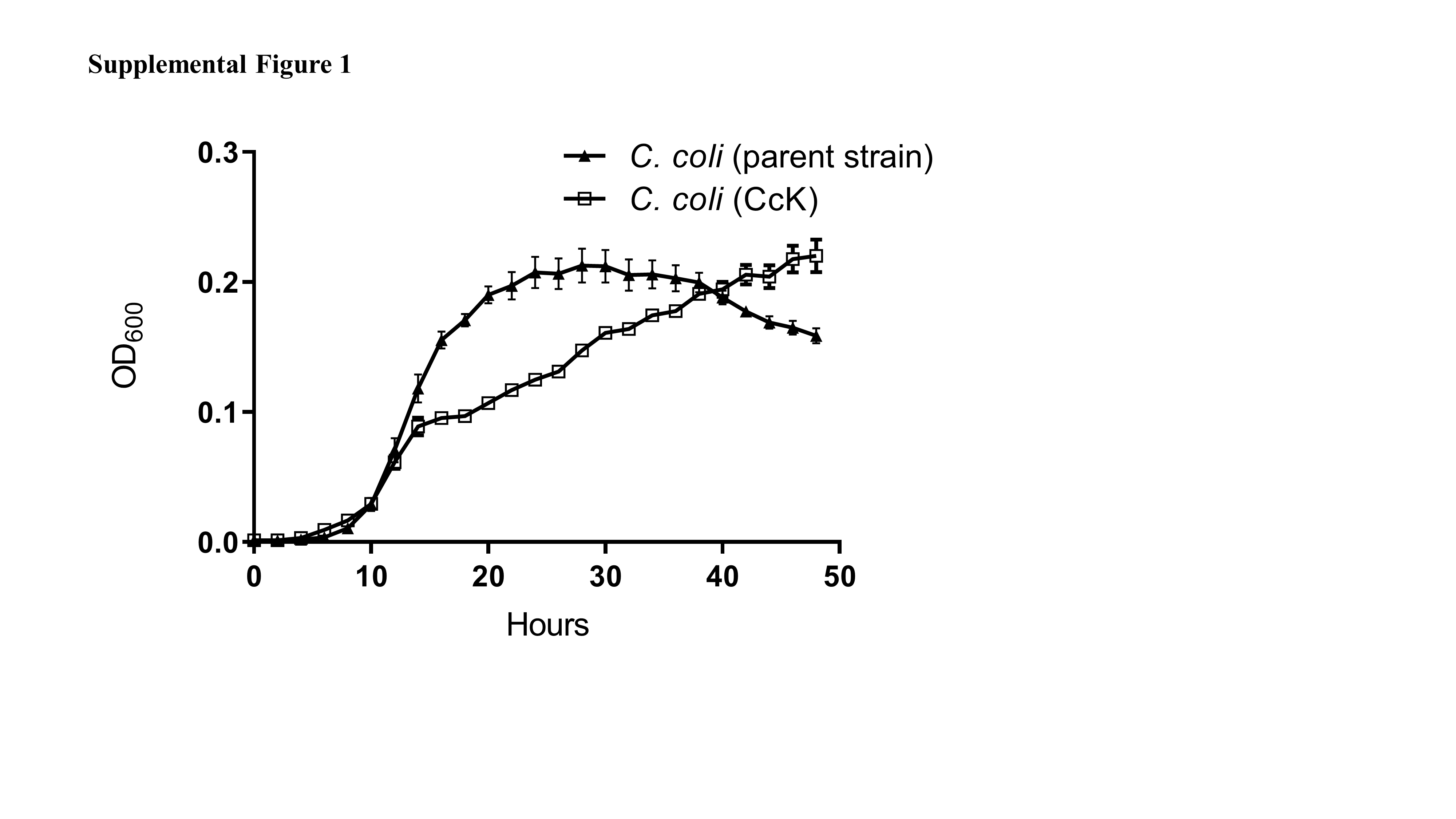

Supplement: Supplementary Figure 1 — Growth characteristics of C. coli wild-type (parent strain) NCTC 80-102, and kanamycin-resistant construct (CcK). Growth curves were performed in Mueller Hinton broth for 48 h at 42°C in a microaerophilic environment (5% O2, 10% CO2, and 85% N2). Data represent the mean ± SEM OD600 of 8 cultures in Bolton's broth measured every 2 h for a total of 48 h with the background value of unincolated media subtracted. [file Image_1.TIF]

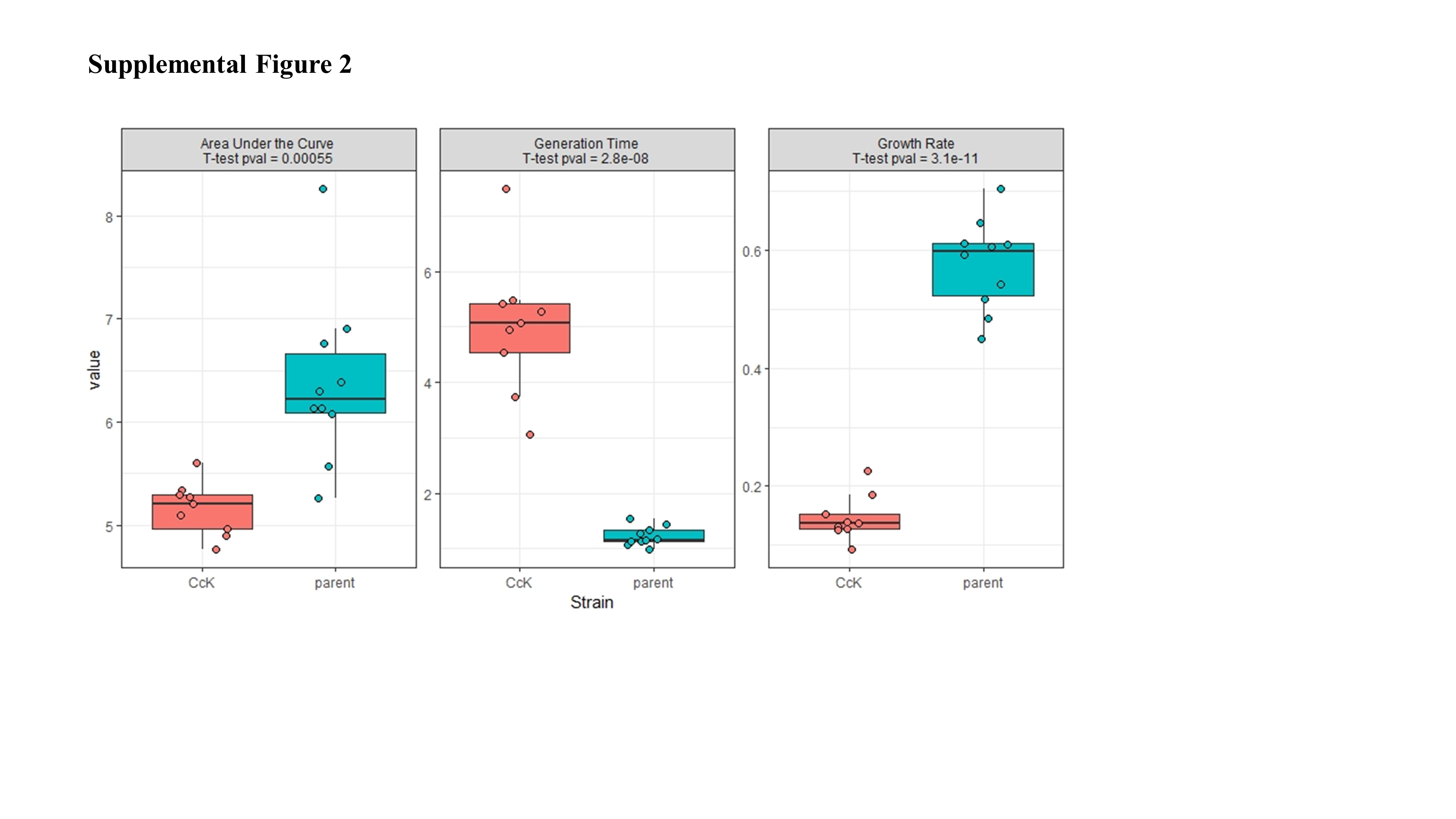

Supplement: Supplementary Figure 2 — Analysis of logistical area under the curve, growth rate and generation time of C. coli wild-type (parent) NCTC 80-102, and kanamycin-resistant construct (CcK). Growth curves were performed in Mueller Hinton broth for 48 h at 42°C in a microaerophilic environment (5% O2, 10% CO2, and 85% N2). Data represent the mean ± SEM logistical area under the curve, growth rate and doubling time of 8 replicate cultures in either broth. Statistical differences in the each variable were determined using the R package growthcurver followed by a post-hoc multiple comparisons test (Tukey). Significant differences (p < 0.05) between different organisms are represented by different letters. [file Image_2.TIF]

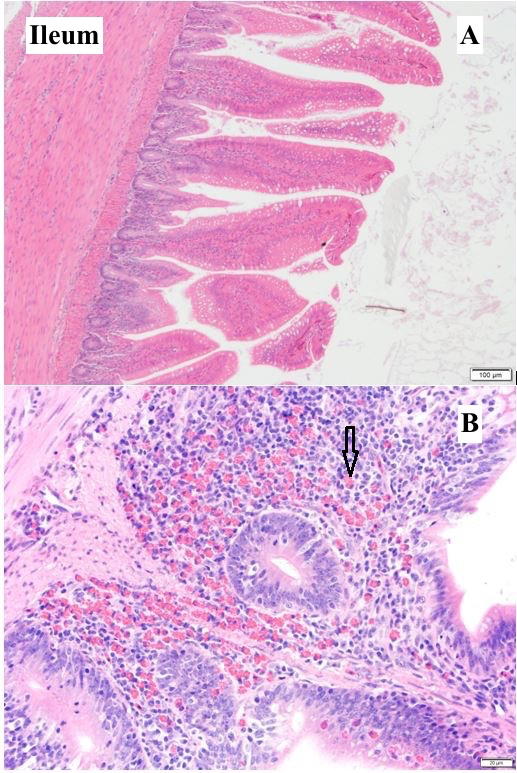

Supplement: Supplementary Figure 3 — Representative Histological of ileal tissues. Representative comparisons are shown for (A) an ileum from a mock-colonized bird and (B) ileum from a CcK-colonized bird, with heterophilic ileitis marked with an arrow. [file Image_3.TIFF]

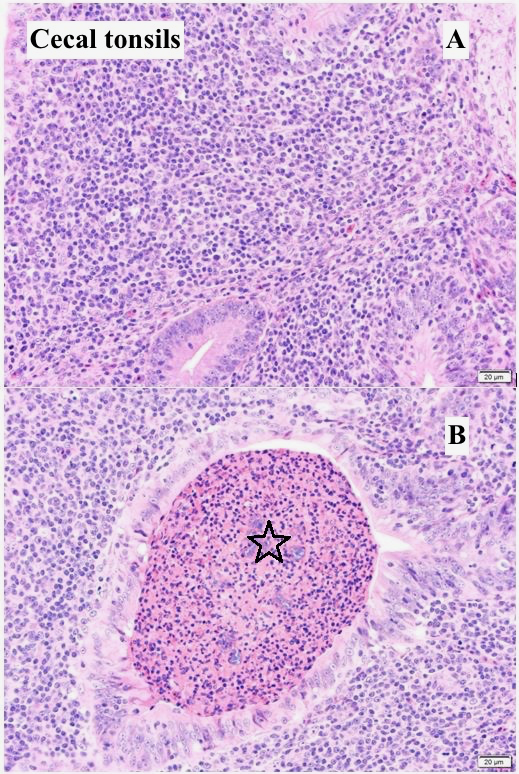

Supplement: Supplementary Figure 4 — Representative Histological of cecal tonsil tissues. Representative comparisons are shown for (A) a cecal tonsil from a mock-colonized bird and (B) cecal tonsil from a CcK-colonized bird, with necrotic cecal crypts with intralesional bacteria marked with a star. [file Image_4.TIFF]

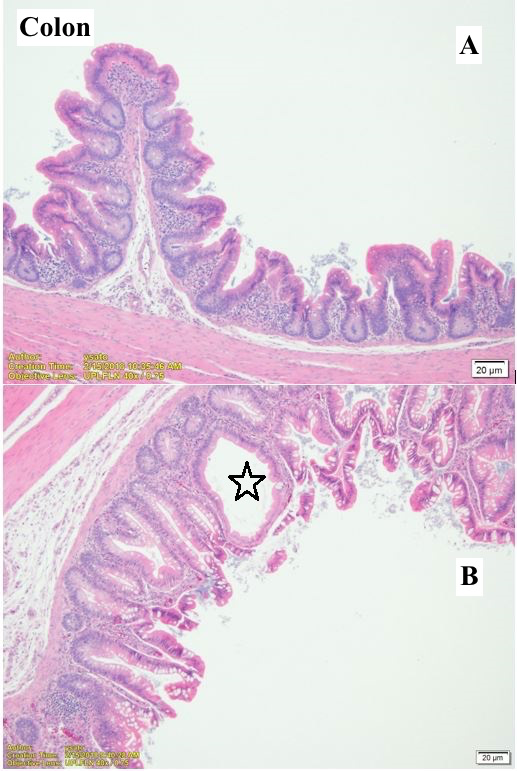

Supplement: Supplementary Figure 5 — Representative Histological of colonic tissues. Representative comparisons are shown for (A) a colonic tissue from a mock-colonized bird and (B) colonic tissue from a CcK-colonized bird, with crypt dilatation/ectasia marked with star. [file Image_5.TIFF]

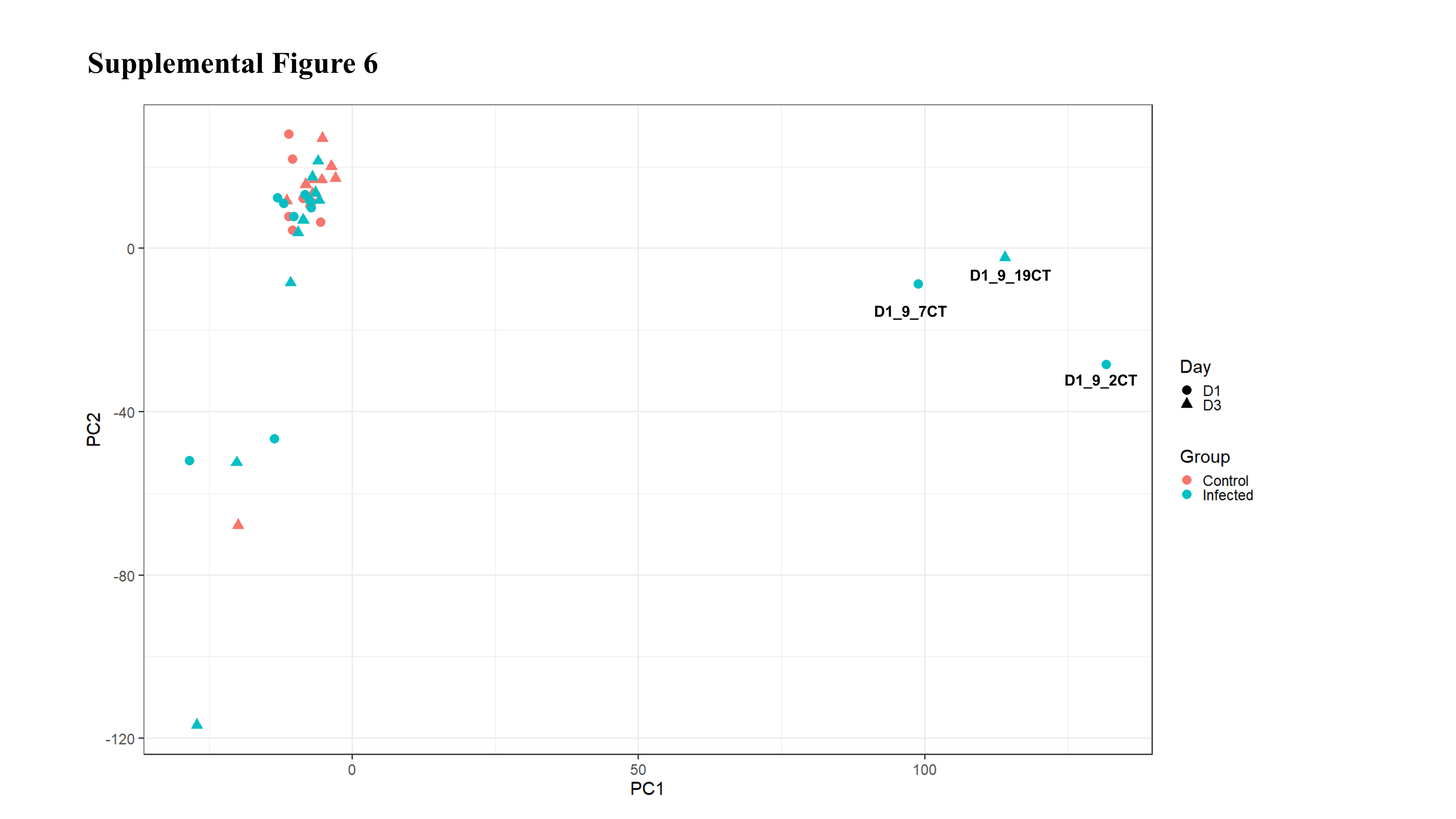

Supplement: Supplementary Figure 6 — PCA analysis of RNAseq samples. [file Image_6.TIFF]
